# Genomics, genetics and breeding of tropical legumes for better livelihoods of smallholder farmers

**DOI:** 10.1111/pbr.12554

**Published:** 2018-04-17

**Authors:** Chris Ojiewo, Emmanuel Monyo, Haile Desmae, Ousmane Boukar, Clare Mukankusi‐Mugisha, Mahendar Thudi, Manish K. Pandey, Rachit K. Saxena, Pooran M. Gaur, Sushil K. Chaturvedi, Asnake Fikre, NPVR Ganga Rao, CV SameerKumar, Patrick Okori, Pasupuleti Janila, Jean Claude Rubyogo, Chigeza Godfree, Essegbemon Akpo, Lucky Omoigui, Stanley Nkalubo, Berhanu Fenta, Papias Binagwa, Michael Kilango, Magdalena Williams, Omari Mponda, David Okello, Mekasha Chichaybelu, Amos Miningou, Joseph Bationo, Dramane Sako, Sory Diallo, Candidus Echekwu, Muhammad Lawan Umar, Richard Oteng‐Frimpong, Haruna Mohammed, Rajeev K. Varshney

**Affiliations:** ^1^ International Crops Research Institute for the Semi‐Arid Tropics (ICRISAT) Nairobi Kenya; ^2^ ICRISAT Bamako Mali; ^3^ International Institute of Tropical Agriculture (IITA) Kano Nigeria; ^4^ International Center for Tropical Agriculture (CIAT) Kampala Uganda; ^5^ ICRISAT Hyderabad India; ^6^ ICAR‐Indian Institute of Pulses Research (IIPR) Kanpur India; ^7^ ICRISAT Addis Ababa Ethiopia; ^8^ ICRISAT Nairobi Kenya; ^9^ ICRISAT Lilongwe Malawi; ^10^ CIAT Arusha Tanzania; ^11^ IITA Lusaka Zambia; ^12^ IITA Kano Nigeria; ^13^ National Agricultural Research Organization (NARO) Namulonge Uganda; ^14^ Ethiopian Institute of Agricultural Research (EIAR) Melkassa Ethiopia; ^15^ Selian Agricultural Research Institute (SARI) Arusha Tanzania; ^16^ Agricultural Research Institute (ARI) Uyole Tanzania; ^17^ ARI Maruku Tanzania; ^18^ ARI Naliendele Tanzania; ^19^ National Semi Arid Resources Research Institute (NaSARRI) Soroti Uganda; ^20^ EIAR Debre Zeit Ethiopia; ^21^ Environmental Institute for Agricultural Research (INERA) Ouagadougou Burkina Faso; ^22^ Institut de l'Economie Rurale (IER) Kayes Mali; ^23^ IER Cinzana Mali; ^24^ Institute for Agricultural Research (IAR) Kano Nigeria; ^25^ Savanna Agricultural Research Institute (SARI) Tamale Ghana

**Keywords:** grain legumes, legume breeding, legume genetics, legume genomics, legume variety adoption, legume variety release, tropical legumes

## Abstract

Legumes are important components of sustainable agricultural production, food, nutrition and income systems of developing countries. In spite of their importance, legume crop production is challenged by a number of biotic (diseases and pests) and abiotic stresses (heat, frost, drought and salinity), edaphic factors (associated with soil nutrient deficits) and policy issues (where less emphasis is put on legumes compared to priority starchy staples). Significant research and development work have been done in the past decade on important grain legumes through collaborative bilateral and multilateral projects as well as the CGIAR Research Program on Grain Legumes (CRP‐GL). Through these initiatives, genomic resources and genomic tools such as draft genome sequence, resequencing data, large‐scale genomewide markers, dense genetic maps, quantitative trait loci (QTLs) and diagnostic markers have been developed for further use in multiple genetic and breeding applications. Also, these mega‐initiatives facilitated release of a number of new varieties and also dissemination of on‐the‐shelf varieties to the farmers. More efforts are needed to enhance genetic gains by reducing the time required in cultivar development through integration of genomics‐assisted breeding approaches and rapid generation advancement.

## 1 INTRODUCTION

Cropping systems intensification and diversification with legumes improve household food, nutrition and income security (Ojiewo et al., 2015). In addition to high protein and micronutrient and oil content, legumes have additional features of soil nitrogen fixation, mineral acquisition from deeper soil layers and addition of organic matter to the soil in form of leaf drops and decaying roots, which contribute to soil health and agro‐ecosystem stability. Besides, legume crops increase and diversify smallholder incomes and hence buffer them from the effects of price fluctuations, and pest and climate‐related production fluctuations.

The optimal ratio between cereals and legumes in the average diet is approximately 2:1, resulting in an amino acid balance that is near‐optimal for human nutrition (Bressani, 1988). Legumes can play much greater role in enhancing nutritional security across the age, gender and economic groups as these crops have much higher protein content of better quality than the major cereals, which increases the biological value of combined protein when consumed together. Besides, grain legumes have higher dietary fibre, mineral micronutrients, phenolic compounds and some vitamins.

The ability of legumes to fix soil nitrogen and improve soil health enhances farm productivity and smallholder incomes, while reducing the high costs of production incurred through exogenous application of inorganic fertilizers. The fixed nitrogen is gradually released from decaying root (and shoot) biomass, thereby improving the soil fertility and making the nitrogen available for the subsequent crop (or intercrop), mostly cereals (Crews & Peoples, 2005). The subsequent cereal crop will give not only higher grain yields for human consumption but also higher stover yields for livestock feed. Legume haulm itself is nutritionally rich as livestock feed which improves let‐down and meat quality in crop livestock‐producing communities. The livestock manure goes back to the crop production fields to improve the soil structure and fertility, and ensures sustainable management land resource. Thus, legumes support the crop‐livestock system that enhances system efficiency and sustainability as well as resilience of smallholder farmers to climate shocks (Ojiewo et al., 2015).

Surplus legume harvest is sold at higher unit prices than cereals to generate family income that can be used to meet household needs such as alternative foodstuff, medicine, school fees among others. Legumes are on high demand especially in India, which is still a net importer (Alene, Coulibaly, & Abdoulaye, 2012). Pigeonpea, for example, has substantial regional and international export potential, and India alone imports 506,000 MT annually (Kumar, 2014). Recently, the East African Grain Council together with the International Trade Centre signed an agreement with India to access its pulses market estimated at US$ 4 billion (Business Daily, Monday, 28 April 2015).

**Figure 1:**
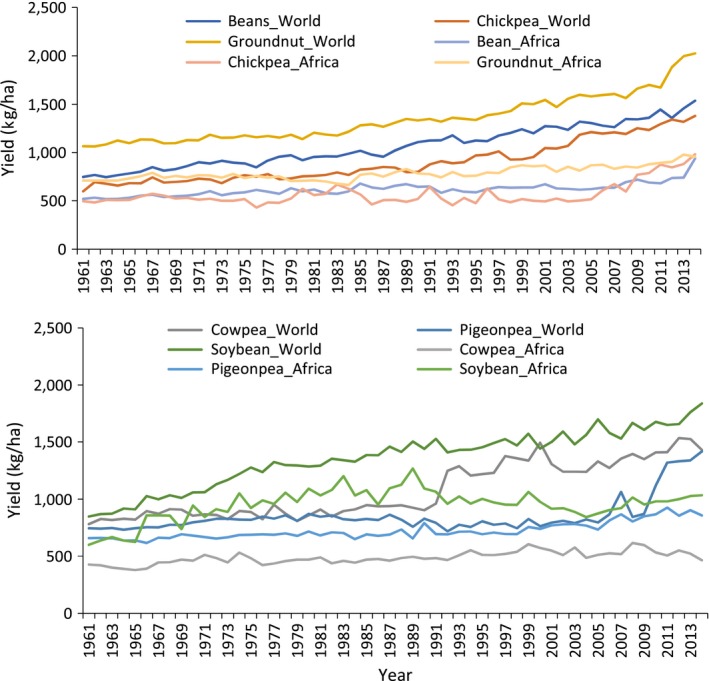
Global and SSA comparative figures on yield increase in selected legumes over the years

Despite their importance, the productivity of legumes in sub‐Saharan Africa (SSA) is very low compared to global averages (Figure 1). Diseases such as Fusarium wilt in chickpea and pigeonpea and rosette in groundnut can cause 100% yield loss. Pest damage, especially during critical growth and development stages, also causes severe yield losses. Abiotic and edaphic constraints, such as salinity, low soil fertility, heat stress and drought—which are the major and common constraints across all countries in the region—reduce legumes yields substantially. Highly variable climate regimes compound the problem. Quality issues such as aflatoxin in groundnuts affect farmers’ incomes due to restrictions in specific export markets besides affecting the health of the consumers.

The physiological mechanisms of drought tolerance in relation to water economy have been studied using high‐throughput phenotyping and well‐characterized mapping populations (e.g., Vadez et al., 2015). It is necessary to further test, identify and deploy molecular markers for drought and/or heat tolerance for selected food legumes through systematic collection of orthologous sequence information of candidate genes, which were reported to have a functional effect on tolerance or resistance to drought and heat in other plants.

A better understanding of host‐plant resistance and diversity in resistance sources and the pathogens is necessary for important soil‐borne diseases. With changes in climatic patterns, changes and shifts in pathogen and pest diversity are likely to become more common. For instance, disease surveillance studies conducted in Uganda resulted in a new root rot pathogen; *Sclerotium rolfsii* being identified as an emerging disease that occurred in most bean fields, causing devastating yield losses (Paparu et al., 2015).

Pests such as pod borers (*Helicoverpa armigera* and *Maruca vitrata*), stem maggots (*Ophiomyia* spp) and bruchids (*Acanthoscelides* spp) cause serious losses in these legumes. Only low levels of resistance to pod borers exist in cultivated germplasm. Therefore, screening of wild gene pools and introgression from wild relatives complemented with transgenic approaches are recommended. Significant advances have been made in breeding for resistance to parasitic pests (*Striga*,* Orobanche, Alectra*) with some success to striga in cowpea, while sources of resistance to *Orobanche* have been identified for lentil and faba bean (Boukar, Kong, Singh, Murdock, & Ohm, 2004; Singh et al., 2006). Efforts are underway to identify sources of resistance to *Alectra* in groundnut and cowpea.

Key quality traits including protein content and micronutrients (iron, zinc, calcium) and oil content are yet to be fully addressed. Exploration for molecular markers for these quality traits is already in progress, and further functional studies using targeted analysis of specific pathways and regulatory genes are recommended. Diagnostic markers for improving groundnut oil quality by increasing oleic acid and decreasing the contents of linoleic and palmitic acids are available (Pandey et al., 2016). Through the HarvestPlus challenge programme, some progress has been made in identifying the diversity that exists in micronutrient (Fe and Zn) grain content for the common bean and in developing breeding lines with higher grain concentrations of these micronutrients. Results on the bioavailability of these micronutrients in human diets are yet to be reported. Further research on exploring the genetic variation for different nutrients is required for biofortifying these legume crops to strengthen fight against malnutrition.

Through the Tropical Legumes Projects I, II and III (TLI, TLII and TLIII), and through the CGIAR Research Program on Grain Legumes (CRP‐GL), International Crops Research Institute for the Semi‐Arid Tropics (ICRISAT), International Institute of Tropical Agriculture (IITA), International Center for Tropical Agriculture (CIAT) and International Center for Agricultural Research in the Dry Areas (ICARDA) have been working with international and national partners on breeding pipelines for respective legume crops with enhanced genetic gains and breeding efficiency for a continuous development of new improved varieties. Intensive and comprehensive research efforts are being made in genetic resource characterization and utilization, trait discovery, prebreeding, forward breeding and variety (product) development as briefly described below with a focus on priority traits to enhance genetic gains in legume crops.

Continuous genetic improvement for enhanced productivity, production, quality and adoption of higher‐yielding cultivars of these grain legumes will enhance their sustainable and timely availability, accessibility and affordability. In addition, improved cultivars of these crops are also more responsive to improved crop management for high productivity, making them increasingly more relevant to reducing hunger in the areas they are traditionally grown and consumed.

## 2 PLANT GENETIC RESOURCE CHARACTERIZATION AND UTILIZATION

Phenotypic and molecular characterization of legume genetic resources held at ICRISAT, CIAT, IITA and ICARDA in collaboration with Generation Challenge Programme (GCP) produced reference sets corresponding to the genetic diversity of several composite collections of chickpea, groundnut, pigeonpea, cowpea, common bean, faba bean and lentil (Glaszmann, Kilian, Upadhyaya, & Varshney, 2010; Gowda, Upadhyaya, Sharma, Varshney, & Dwivedi, 2013; Mahalakshmi, Ng, Lawson, & Ortiz, 2006; Upadhyaya et al., 2008, 2011). These reference sets have been the subject of further high‐throughput genotyping and next‐generation sequencing including whole‐genome sequences (Varshney et al., 2012; Varshney, Song et al., 2013; Varshney, Gaur et al., 2013; Varshney, Thudi et al., 2014; Varshney, Mohan et al., 2014; Varshney, Pandey et al., 2014; Varshney et al., 2017; Bertioli et al., 2016; Chen et al., 2016).

The genetic structure, diversity and allelic richness in a composite collection of chickpea was studied using single‐sequence repeat (SSR) markers, and a reference set of 300 accessions was formed in 2008 (Upadhyaya et al., 2008). Phenotyping of mini core collection of chickpea has identified traits of agronomic importance including drought, salinity, high temperature and herbicide tolerance, resistance to Fusarium wilt, Ascochyta blight, Botrytis grey mould and pod borer (Upadhyaya et al., 2013). Kebede (2013) used single‐nucleotide polymorphism (SNP) markers to characterize Ethiopian GenBank collection of chickpea and developed a core collection of 158 from 1002 accessions.

Phenotyping of pigeonpea composite collection for 16 quantitative and 16 qualitative traits resulted in the identification of promising diverse accessions for the four important agronomic traits: early flowering (96 accessions), high number of pods (28), high 100‐seed weight (88) and high seed yield/plant (49). A reference set comprising 300 most diverse pigeonpea accessions was constituted based on SSR genotyping data in 2011 (Upadhyaya et al., 2011). This reference set has been characterized through whole‐genome resequencing (WGRS) and provided detailed phylogenetic relationships among accessions (Varshney et al., 2017). Additionally analysis of WGRS data has provided several genomic regions that were likely targets of domestication and breeding, and associations between several candidate genes and agronomically important traits (Varshney et al., 2017).

A reference set consisting of 300 genetically most diverse accessions of groundnut has been developed. This reference set captured 466 (95%) of the 490 composite collection alleles, representing diversity from the entire spectrum of composite collection. Further phenotyping and marker‐based profiling of the groundnut reference set have revealed traits of economic importance and identified accessions for beneficial traits such as early‐maturing groundnut (90 days) with high pod yield or those with large variability in pod/seed characteristics, oil content and high oleic acid, and grain Fe and Zn contents; tolerance to drought, salinity and low temperature; resistance to root‐knot nematode and early and late leaf spot (Gowda et al., 2013). The reference sets are ideal for allele mining, association genetics, mapping and cloning gene(s), and in applied breeding for the development of broad‐based elite breeding lines/cultivars with superior yield and enhanced adaptation to diverse environments. A comprehensive genomewide association study (GWAS) was conducted on this diverse set leading to identification of >500 marker–trait associations for several agronomically important traits including pod yield under water‐limited conditions (Pandey et al., 2014). Further efforts are underway to deploy next‐generation sequencing (NGS)‐based high‐density genotyping for conducting high‐resolution GWAS for several traits of importance in groundnut.

The cowpea reference set comprises about 374 accessions representing the entire genetic diversity of cowpea present in IITA Genetic Resource Center. This reference set was derived from the core collection of 2062 accessions constituted from geographical, agronomical and botanical descriptors (Mahalakshmi et al., 2006). The diversity in the reference set was similar to that of the entire collection, and correlated traits that may be linked were also preserved in the core collection and the reference set. This set is a critical resource for breeders and other plant scientists for further exploitation of the cowpea germplasm for improvement. For example, new sources of resistance to *Striga* were identified recently from this set (Omoigui et al., 2017). In addition, a reference kit of 20 SSRs with primer sequence list and SNP data for association mapping studies have been generated from this set.

## 3 TRAIT DISCOVERY

New genetic and genomic resources are just becoming available, which allow breeding to be taken to the next level. In the case of pigeonpea, comprehensive application of genomics for crop improvement began with the availability of large amount of markers, mapping populations and draft genome sequence (Varshney et al., 2012). In the draft genome, a total of 48,680 genes were predicted and also showed the potential role that certain gene families, for example, drought tolerance‐related genes, have played throughout the domestication of pigeonpea and the evolution of its ancestors (Varshney et al., 2012). This reference genome sequence has been the base of resequencing based mapping studies (Saxena et al., 2017; Singh et al., 2015), which has enabled researchers to develop trait‐associated markers quickly.

The chickpea genome sequencing project reported ~738‐Mb draft whole‐genome shotgun sequence of ‘CDC Frontier’, a kabuli chickpea variety, which contains an estimated 28,269 genes (Varshney, Song et al., 2013). Resequencing and analysis of 90 cultivated and wild genotypes from 10 countries identified targets of both breeding‐associated genetic sweeps and breeding‐associated balancing selection. In addition, parental genotypes of different mapping populations available at ICRISAT provided several million SNPs, indels and structural variations that can be used for trait dissection (Thudi, Chitikineni et al., 2016). Further, resequencing of 129 released varieties provided insights into temporal and special trends in diversity as well as the effect of breeding diversity over decades (Thudi, Khan et al., 2016). The SNPs identified in different studies were used to develop 56K SNP array, which find broader application in genetic and genomic studies for chickpea improvement (Roorkiwal et al. 2017). Candidate genes for disease resistance and agronomic traits that distinguish the two main market classes of cultivated chickpea—desi and kabuli—were reported.

The International Peanut Genome Initiative (IPGI) sequenced the whole genome of *Arachis duranensis* and *A. ipaensis*, the diploid ancestors of cultivated peanut, *A. hypogaea*, an allotetraploid with closely related subgenomes of a total size of ~2.7 Gb (Bertioli et al., 2016). Comparison of the DNA sequences of one of the wild species with the cultivated groundnut showed that they are 99.96% identical, with *A. duranensis* and *A. ipaensis* genomes being similar to *A. hypogaea's* A and B subgenomes. The whole‐genome sequences were used to identify candidate disease resistance genes, to guide tetraploid transcript assemblies and to detect genetic exchange between cultivated peanut subgenomes (Chen et al., 2016). The downstream utilization of genome sequence will lead to better groundnut varieties with enhanced pod and oil yield, greater resistance to diseases, tolerance to drought and heat and better oil quality. The *A. duranensis* genome provides a major source of candidate genes for fructification, oil biosynthesis and allergens, expanding knowledge of understudied areas of plant biology and human health impacts of plants (Chen et al., 2016). These genome sequences also provide millions of structural variations that can be used as genetic markers for the development of improved peanut varieties through genomics‐assisted breeding. Of these structural variations, SNP and SSRs have been the preferred marker types, and therefore, a high‐throughput genotyping array with >58K highly informative genomewide SNPs was developed (Pandey et al., 2017) along with thousands of SSRs for use in genetics and breeding studies (Zhao et al., 2017).

A draft genome sequence of mungbean has also been constructed to facilitate genome research into the subgenus *Ceratotropis*, which includes several important dietary legumes in Africa, and to enable a better understanding of the evolution of leguminous species (https://www.nature.com/articles/ncomms6443). The *de novo* assembly of a tetraploid *Vigna* species (*V. reflexo–pilosa* var. *glabra*) provided genomic evidence of a recent allopolyploid event (Kang et al., 2014). The species tree was constructed using *de novo* RNA‐Seq assemblies of 22 accessions of 18 *Vigna* species and protein sets of *Glycine max*. The present assembly of *V. radiata* var. *radiata* will facilitate genome research and accelerate molecular breeding of the subgenus *Ceratotropis*.

Early initiative on the development of cowpea genomic resources performed the sequencing of the gene‐rich region of the cowpea genome (called the genespace) recovered using methylation filtration technology and providing annotation and analysis of the sequence data. About 298,848 cowpea genespace sequences (GSS) isolated by methylation filtering of genomic DNA were used to develop a database (Chen, Laudeman, Rushton, Spraggins, & Timko, 2007). This database consisted of GSS annotation and comparative genomics knowledge base, GSS enzyme and metabolic pathway knowledge base, and GSS SSRs knowledge base for molecular marker discovery. Timko et al. (2008) reported that more than 250,000 genespace sequence reads (GSRs) were generated, assembled, annotated by basic local alignment search tool (BLAST) homology searches of public databases (http://cowpeagenomics.med.virginia.edu/) and analysed using various domain and gene modelling tools. A total of 41,260 GSR assemblies and singletons were annotated, of which 19,786 have unique GenBank accession numbers. Cowpea genome sequences of an estimated 97% of all cowpea genes are available in a 60X genome coverage assembly from BLAST server of University of California, Riverside (UC Riverside).

Using resequencing of 60 wild individuals and 100 landraces of common bean from the genetically differentiated Mesoamerican and Andean gene pools of the common bean as a reference genome, Schmutz et al. (2010) confirmed two independent domestications from genetic pools that diverged before human colonization. Less than 10% of the 74 Mb of sequence putatively involved in domestication was shared by the two domestication events (https://www.nature.com/articles/ng.3008). The findings by Schmutz et al. (2010) provide information on regions of the genome that have undergone intense selection, either during domestication or early improvement, and thus provide targets for future crop improvement efforts.

The 1.1 Gb genome sequence of the palaeopolyploid soybean was reported in 2010 and 46,430 protein‐coding genes were predicted, 78% of which occur in chromosome ends (Schmutz et al., 2010). Soybean genome is reported to have undergone two duplications events with about 75% of the genes present in multiple copies. Subsequently, gene diversification and loss, and numerous chromosome rearrangements took place (Schmutz et al., 2010). These results are expected to facilitate the identification of the genetic basis of many soybean traits and accelerate soybean variety development.

With these advances in genomic information and breeding support tools, there exists significant potential for improving genetic gains even under low‐input agriculture through the implementation of systematic breeding, tapping into the available natural diversity and the use of modern tools and technologies.

## 4 PREBREEDING

The wild species and landraces are valuable sources of new genes and alleles, particularly for resistance to biotic and abiotic stresses. These have largely remained under‐utilized due to barriers to interspecific hybridization, lack of evaluation data on specific traits, lack of ready access to advanced breeding tools, linkage drag due to negative alleles in regions flanking the desirable alleles and the tendency to emphasize short‐term outputs in breeding projects.

In chickpea, two closely related species, *Cicer reticulatum* and *C. echinospermum*, have been used for widening the genetic base by introgressing genes for resistance/tolerance to Phytophthora root rot, cyst nematode (*Heterodera ciceri*), root‐lesion nematode (*Pratylenchus* spp.), pod borer (*Helicoverpa armigera*), Ascochyta blight, Botrytis grey mould and low temperatures (Gaur et al., 2010). Wild *Cajanus* species have been effectively exploited in developing cytoplasmic male sterility (CMS) systems, which made commercial hybrids possible (Saxena et al., 2015). In *Phaseolus* beans, the cultivated species of the secondary (*P. coccineus* and *P. dumosus*) and the tertiary (*P. acutifolius*) gene pools have been used for the improvement of common bean (*P. vulgaris*) (Porch et al., 2013). In lentil, genes for anthracnose and wilt resistance and drought tolerance have been introgressed in the elite pool from *L. lamottei* (Fiala, Tullu, Banniza, Seguin‐Swartz, & Vandenberg, 2009).

Utilization of wild *Arachis* species following interspecific hybridization has resulted in the development of many elite germplasm lines and cultivars with improved levels of resistance to disease and insect pests (Sharma, Upadhyaya, Varshney, & Gowda, 2016). The development and utilization of synthetic amphidiploid cultivars such as ‘TxAG 6’ with large genetic variation have made possible the transfer of resistance genes from wild *Arachis* species into cultivated groundnut variation (Simpson, Starr, Nelson, Woodard, & Smith, 1993).

Several wild accessions of *Vigna* species such as *V. vexillata*,* V. davyi*,* V. oblongifolia* and *V. luteola* showed good levels of resistance to insect pests that devastate cowpea (Fatokun, 2002). Efforts are underway to screen some wild relatives of cowpea including *V. unguiculata ssp. rhomboidea* and *V. unguiculata ssp. protracta var. kgalagadiensis* for sources of resistance to key production constraints. These wild relatives may share the same primary gene pool with cowpea (Fatokun, Perrino, & Ng, 1997) and therefore should be cross‐compatible to cultivated cowpea.

## 5 DIAGNOSTIC MARKERS FOR USE IN BREEDING

A backcross process is accelerated by coupling phenotypic selection with marker‐assisted selection for the genetic background of the recurrent parent, a process called “background selection” or “donor genome elimination,” and the most recent breeding advances are included into the backcross breeding programme (Bliss, 2009).

Using polyacrylamide gel electrophoresis, Ouedraogo, Ouedraogo, Gowda, and Timko (2012) isolated an amplified fragment length polymorphism (AFLP) fragment, E‐ACT/M‐CAA524, tightly linked to the *Striga gesnerioides* race 1 (SG1) resistance gene *Rsg‐2‐1* in cowpea (*V. unguiculata* L.), cloned and determined its nucleotide sequence. The authors designed two sequence‐specific primers and used them to isolate similar fragments from genomic DNA of different cowpea lines by polymerase chain reaction (PCR) amplification. The primers amplified a ~500‐bp fragment (sequence‐characterized amplified region (SCAR) marker designated as 61R) that was present in the resistant parent “TVU‐14676,” absent in susceptible parent “IT84S‐2246,” and segregated with the resistance phenotype in an F2 population, derived from a cross of these two genotypes. They then used the primers to isolate a fragment similar to 61R from another *S. gesnerioides* resistant line “KVX 61‐1” and used the sequence of this fragment to design a new combination of primers that developed a second SCAR marker, designated as “61R‐M2.” The three markers, E‐ACT/M‐CAA524, 61R and 61M2, are linked to each other by 0.6 centimorgans (cM) and are available for use in introgression of *Striga* resistance into susceptible cowpea lines.

Quantitative trait loci for heat tolerance in cowpea were reported by Lucas et al. (2013) from studies based on varieties ‘CB 27’ and ‘IT82E‐18’. Five regions, representing 9% of the cowpea genome, were identified to explain 11.5%–18.1% of the phenotypic variation for heat tolerance and tagged with 48 transcript‐derived SNP markers. Favourable haplotypes were donated by CB27 for four of these regions while IT82E‐18 was the source of tolerance explained by the fifth QTL.

Current research seeks to leverage the cowpea genespace sequence data and modern molecular‐based technologies, in combination with conventional breeding strategies to increase the speed at which a greater number of superior‐performing, well‐adapted cowpea varieties containing pyramided agronomic productivity, disease and pest resistance traits can be delivered to local farmers. SNPs associated with drought tolerance, bacterial blight and *Striga* resistance in cowpea were identified at UC‐Riverside and validated at IITA (Beebe et al., 2016).

Under TLII project, for example, marker‐assisted backcrossing was initiated to introgress *Striga* resistance into well‐adapted released varieties but susceptible to *Strig*a. BC_2_F_1_ were generated. Over 1,200 germplasm lines were evaluated in the field for their drought tolerance and 20 lines with enhanced drought tolerance were identified and crossed to existing breeding lines with farmer and consumer preferred traits. Over 200 populations segregating for drought tolerance and resistance to *Striga* were generated. (Boukar et al., 2016).

In common bean, potyviral resistance derived from cultivars of *Phaseolus vulgaris* carrying *bc‐3* has been reported to be associated with the homozygotic presence of a mutated *eIF4E* allele (Naderpour, Sogaard‐Lund, Larsen, & Johansen, 2010). F_2_ plants homozygous for the eukaryotic translation initiation factor, *eIF4E* mutant allele, were found to display at least the same level of resistance to bean common mosaic necrosis virus (BCMNV) as the parental resistant genotype. The resistance sources of bean common bacterial blight (CBB) GN#1 sel 27, XAN 159 and OAC 88‐1 each contribute two independent quantitative trait loci (QTLs) with major effect for CBB resistance. SCAR linked with five of the six QTLs are available for marker‐assisted selection (Geunhwa et al., 1997; Miklas et al., 2000). A random amplified polymorphic DNA (RAPD) marker (OPH18_1200C_) linked in resistance to race 73 of *Colletotrichum lindemuthianum* causing anthracnose in beans was identified in a BC_3_F_2_:_3_ population derived from crosses between Ruda and G 2333 (Alzate‐Marin et al., 2001). A RAPD molecular marker OPAS13_950C_, previously identified as linked to gene Co‐4^2^ (Young, Melotto, Nodari, & Kelly, 1998), was also amplified in this population. Cosegregation analyses showed that these two markers are located at 5.6 (OPH18_1200C_) and 11.2 (OPAS13_950C_) cM of the Co‐4^2^ gene. DNA amplification of BC_1_F_2_:_3_ plants resistant to race 89 with RAPD marker OPAB_450C_, previously identified as linked to gene Co‐5, indicated that this gene is present in this population (Alzate‐Marin et al., 2001). Keller et al. (2015) reported three QTL regions responsible for angular leaf spot (ALS) resistance, including one major QTL on chromosome Pv04 at 43.7 Mbp explaining over 75% of the observed variation for ALS resistance. They evaluated 153 F4, 89 BC_1_F_2_ and 139 F_4_/F_5_/BC_1_F_3_ descendants with markers in the region of the major QTL and delimited the region to 418 kbp harbouring 36 candidate genes of which 11 serine/threonine protein kinases arranged in a repetitive array constitute promising candidate genes for controlling ALS resistance. They developed SNP markers cosegregating with the major QTL for ALS resistance for use in marker‐assisted introgression of ALS resistance into susceptible bean varieties.

Many common bean lines in the pipeline carry resistance to BCMNV, anthracnose and ALS. Populations of recombinant inbred lines (RILs) have been developed for yield, drought traits and associated biotic constraints. While it has been a challenge to find consistent QTL for yield under drought, focusing on the trait of pod harvest index (PHI) has been more promising, and some candidate QTLs have been validated through additional phenotyping (Beebe et al., 2016). Through collaboration with the United States Department of Agriculture (USDA), CIAT accessed sequences of common bean SNPs, and through the TL I project, they were converted to the KBioscience Competitive allele‐specific polymerase chain reaction (KASPar) platform.

SNP markers for major disease resistance genes—bean common mosaic necrosis virus (BCMNV), CBB, bruchids and ALS—were developed, and markers of other classes (SCARs, SSRs) were also converted to a SNPs for ready use through the SNP platform (Beebe et al., 2016). To date, this platform for key traits (CBB, root rot, bruchids, etc.) is being utilized for breeding purposes (Beebe et al., 2016). While it has been a challenge to find consistent QTL for yield under drought, some candidate QTL for disease resistance have been validated through additional phenotyping and fine mapping (Keller et al., 2015). A multiparent advance generation intercross (MAGIC) developed using eight parents with various elite traits (seed type, abiotic stress, drought adaptation, yield potential, earliness and biotic stress) is being phenotyped to identify additional QTLs for drought and cooking time. A marker‐assisted recurrent selection (MARS) population was also developed (Beebe et al., 2016).

In chickpea, Varshney, Thudi et al. (2014) reported 45 robust main‐effect QTLs (M‐QTLs) explaining up to 58.20% phenotypic variation and 973 epistatic QTLs (E‐QTLs) explaining up to 92.19% phenotypic variation for several target traits. Nine QTL clusters containing QTLs for several drought tolerance traits were identified among which, one cluster harbouring 48% robust M‐QTLs for 12 traits and explaining about 58.20% phenotypic variation present on “CaLG04” was referred to as *QTL‐hotspot* (Varshney, Thudi et al., 2014). This genomic region containing seven SSR markers (ICCM0249, NCPGR127, TAA170, NCPGR21, TR11, GA24 and STMS11) has further been split into two subregions namely *QTL‐hotspot_a* (15 genes) and *QTL‐hotspot_b* (11 genes) with a total of 25 genes, of which four promising candidate genes having functional implications on the effect of QTL‐hotspot for drought tolerance in chickpea were identified (Kale et al., 2015).

Sabbavarapu et al. (2013) reported two novel QTLs which explained 10.4%–18.8% of phenotypic variation for resistance to race 1 of Fusarium wilt (FW) caused by *Fusarium oxysporum* f. sp. *ciceris* and six QTLs explaining up to 31.9% of phenotypic variation for resistance to Ascochyta blight (AB) caused by *Ascochyta rabiei* in chickpea. One major QTL, explaining 31.9% phenotypic variation for AB resistance, was identified, which, together with the two novel QTLs for FW resistance, are important tools for pyramiding for resistance to the two diseases. Major genomic regions for salinity tolerance (Pushpavalli et al., 2015), flowering time (Mallikarjuna et al., 2017) and vernalization (Samineni, Kamatam, Thudi, Varshney, & Gaur, 2016) were also reported. The SNP markers identified the QTL regions used for developing the diagnostic markers for early generation selection for traits like drought, FW and AB.

In groundnut, linked markers for resistance to rust, late leaf spot (LLS), root‐knot nematode and oil quality (high oleic acid and low linoleic and palmitic acid) are available for use in breeding (Varshney et al., 2013, Pandey et al., 2016). The successful effort was made through genetic mapping and conducting QTL analysis for rust and LLS resistance in a recombinant inbred line (RIL) population (TAG 24 ×  GPBD 4). This population was phenotyped for >10 seasons, which resulted in identification of one major QTL for rust resistance and two major QTLs on LLS resistance (Sujay et al., 2012). These QTLs explained up to 83% phenotypic variation, and therefore, linked markers were deployed for use in marker‐assisted backcrossing successfully (Varshney, Pandey et al., 2014). Similarly, the linked markers were developed for oil quality and root‐knot nematode and were exploited in developing improved groundnut genotypes.

## 6 TRAIT DEPLOYMENT FOR VARIETY DEVELOPMENT, RELEASE AND ADOPTION

In chickpea, MAGIC populations were developed using eight parents and making 28 two‐way crosses, 14 four‐way crosses and 78‐way crosses. Over 1,100 MAGIC lines have been developed (Gaur et al., 2016). Breeding lines are also being developed through MARS. Besides, the *QTL‐hotspot* has been transferred through MABC into chickpea varieties such as ‘JG 11’ and Chefe from the donor parent ICC 4958. Foreground selection with up to three SSR markers namely TAA170, ICCM0249 and STMS11 and background selection with up to 10 AFLP primer combinations was undertaken. Root trait phenotyping of these introgression lines has shown higher rooting depth (RDp, average 115.21 ± 2.24 cm), better root length density (RLD, average 0.41 ± 0.20 cm cm‐3), higher root dry weight (RDW, average 1.25 ± 0.08 g cyl‐1) as compared to both recurrent and donor parents (Varshney, Gaur et al., 2013). Mean yield advantages of between 11 and 24% have also been recorded. Introgression lines in the genetic background of JG 11 and ICCV 10 were also evaluated during 2016–17 at different locations in All India Varietal Trials for their possible release as improved varieties. In addition, introgression lines were also developed in the genetic backgrounds of KWR 108, DCP 92‐3 and Pusa 362 by ICRISAT partners in India. The introgression of this genomic region into several elite cultivars in Africa (Ejere, Arerti in Ethiopia; ICCV 95423 and ICC 97105 in Kenya) also enhanced yields in chickpea (Gaur et al., 2016).

Another example of success in chickpea is MABC targeting *foc1* locus and two QTL regions, ABQTL‐I and ABQTL‐II, to introgress resistance to FW and AB, respectively, in variety C 214 (Varshney, Mohan et al., 2014). Foreground selection was conducted with six markers (TR19, TA194, TAA60, GA16, TA110 and TS82) linked to *foc1* in the cross C 214 × WR 315 (FW resistant). Eight markers (TA194, TR58, TS82, GA16, SCY17, TA130, TA2 and GAA47) linked with ABQTL‐I and ABQTL‐II in the cross C 214 × ILC 3279 (AB resistant) were used. Background selection in both crosses was employed with evenly distributed 40 (C214 × WR 315) to 43 (C 214 × ILC 3279) SSR markers in the chickpea genome to select plant(s) with higher recurrent parent genome recovery. With three backcrosses and three rounds of selfing, 22 BC_3_F_4_ lines were generated for C 214 ×  WR 315 cross and 14 MABC lines for C 214 × ILC 3279 cross. Phenotyping of these lines has identified three resistant lines (with 92.7%–95.2% RPG) to race 1 of FW, and seven resistant lines (with 81.7%–85.40% RPG) to AB that are ready for multilocation adaptation trials, variety release and adoption. Introgression lines resistant to FW were also developed in the genetic background of ‘Annigeri‐1’, ‘JG 72’ and ‘Pusa 256’ (Pratap et al., 2017).

Suitable varieties of chickpea were identified with a high yield potential combined with market‐preferred grains and tolerance to biotic (FW, AB, pod borer resistance) and abiotic stresses (drought and heat tolerance). After preliminary evaluation in Kenya, elite materials were shared with the NARS programmes in Ethiopia, Tanzania and Kenya. A total of 27 varieties were released in ESA (Kenya—9, Tanzania—4, Ethiopia—14) since 2007. Adoption of these varieties and accompanying integrated crop management practices in Ethiopia contributed to increased chickpea productivity from 550 kg/ha in 1993 to 1,913 kg/ha in 2014 and total production from 168,000 t on about 109,000 ha to 458,682 t on about 240,000 ha in 2014 (CSA, 2015). The change in production is about 173% over 2003 base figures, and both gains in area (120%) and productivity (248%) have contributed to these remarkable increases. The estimated level of chickpea variety adoption now is between 25 and 30% with significant impact on increased household income and reduced poverty (Verkaart et al., 2017).

Using genetic and genomic resources, a number of economically important traits have been mapped in pigeonpea. Markers associated with FW (Singh et al., 2015), sterility mosaic disease (SMD) (Singh et al. 2015), different agronomic traits related to plant type and earliness (Kumawat et al. 2017), cytoplasmic male sterility (CMS) (Sinha et al. 2015) and hybrid purity assessment (Saxena et al. 2010; Bohra et al. 2011) have been identified. Due to long generation cycle in pigeonpea, the full potential of genomics‐assisted breeding (GAB) has not been realized till recent past. However, a number of GAB programmes have been initiated recently for introducing or combining FW and SMD resistance in targeted varieties/lines. As a result, few promising lines with higher FW/SMD resistance and yield have been promoted in the trials required for varietal release in India (Saxena et al., 2016).

In groundnut, the MABC approach was deployed for pyramiding high oleic acid and nematode resistance (Chu et al. 2011), rust resistance (Varshney, Pandey et al., 2014) and oil quality (Janila, Pandey, Manohar et al., 2016). Chu et al. (2011) deployed cleaved amplified polymorphic sequence (CAPS) markers for selecting mutant alleles of *ahFAD2A* and *ahFAD2B* genes to increase high oleic acid and SSR markers for enhancing nematode resistance. ICRISAT together with its national partners successfully validated linked markers for rust resistance and deployed MABC approach for improving rust resistance in three popular varieties (ICGV 91114, JL 24 and TAG 24) using one dominant (IPAHM103) and three codominant (GM2079, GM1536, GM2301) markers (Varshney, Pandey et al., 2014). These improved introgression lines have shown >40% yield superiority over their respective recurrent parent in addition to enhanced resistance (Janila, Pandey, Manohar et al., 2016). Three of these lines are being promoted in national evaluation system of India for next round of evaluation and release. In addition, three popular varieties were improved for oil quality using MABC and MAS approach by deploying allele‐specific molecular markers (Janila, Pandey, Shasidhar et al., 2016). These improved lines have now been included in the national evaluation system of India for further evaluation and release.

**Figure 2:**
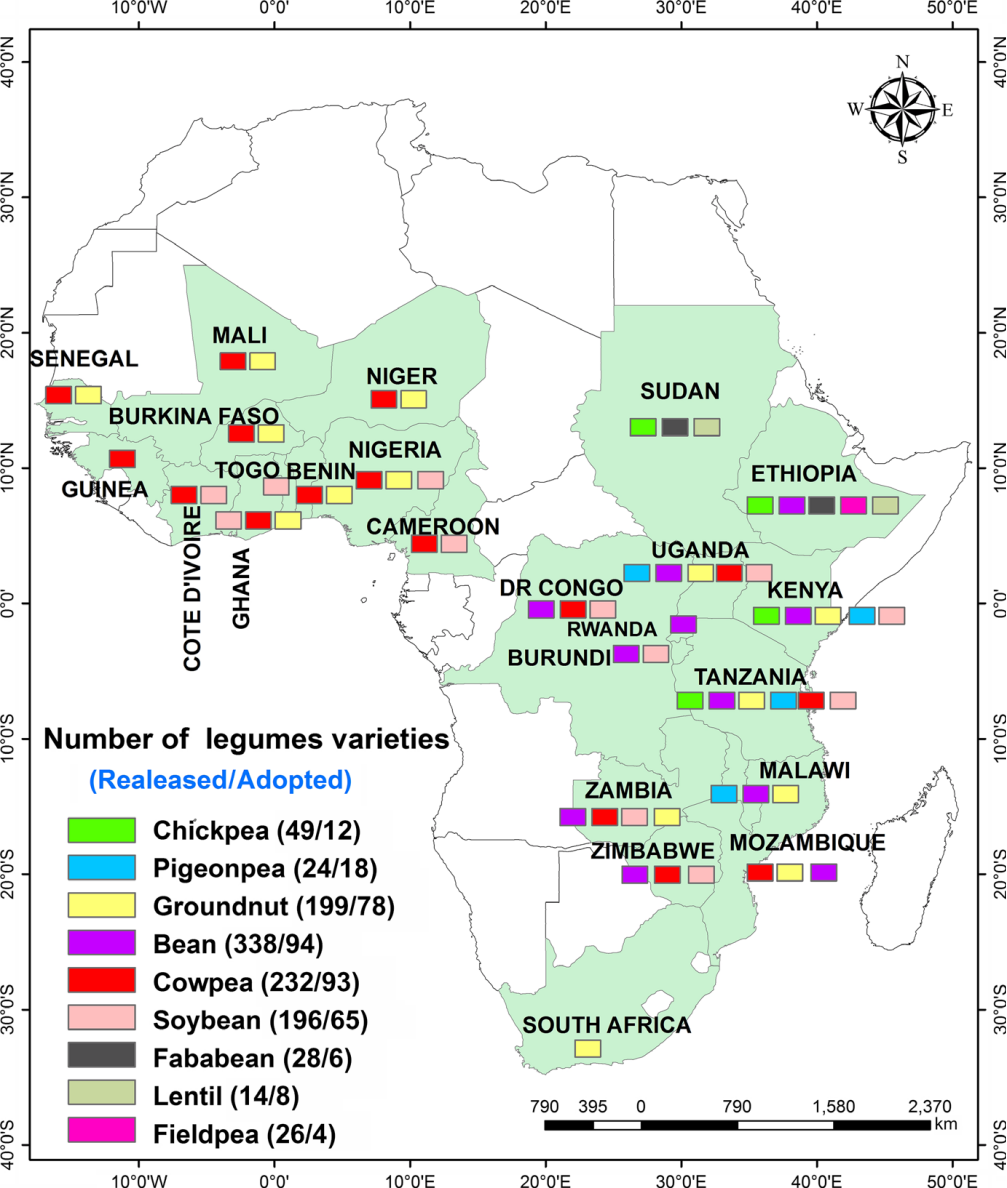
Variety release and adoption as summarized by the CGIAR DIIVA (Diffusion and Impact of Improved Varieties in Africa) project data on selected crops in sub‐Saharan Africa (http://www.asti.cgiar.org/diiva)

Between 2007 and 2017, a total of 37 farmer‐preferred groundnut varieties with early maturity, groundnut rosette disease (GRD) resistance, high yield and other traits were released in different countries, that is Uganda (10), Senegal (6), Mozambique (5), Tanzania (8), Niger (4), Ghana (4) and Nigeria (3) (Figure 2). In Tanzania, adoption of improved varieties and accompanying integrated crop management practices contributed to increased groundnut productivity from 724 kg/ha in 2008 to 1,010 kg/ha in 2014 and total production from 340,770 t on about 470,670 ha to 1,635,335 t on about 1,619,500 ha in 2014 (FAOSTAT, 2014). The change in production is about 480% over 2008 base figures, and both gains in area (244%) and productivity (39%) have contributed to these remarkable increases. The estimated level of improved groundnut variety adoption now is estimated at 19% nationally before correction through DNA variety fingerprinting data inclusion.

In Nigeria, seven cowpea varieties were released with grain yield ranging between 50 and 71% above the local check. These varieties are also drought tolerant, resistant to striga and better adapted to the dry savannah regions. In Mali, four improved cowpea lines (IT97K‐499‐35, IT93K‐876‐30, CZ1‐94‐23‐1 and CZ11‐94‐5C) were released. Adoption of improved varieties and accompanying integrated crop management practices in Nigeria contributed to increased cowpea productivity from 650 kg/ha in 1991 to 1,100 kg/ha in 2013 and total production from 1,372,333 MT on about 2,152,580 ha to 3,971,480 MT on about 3,591,933 ha in 2013 (FAOSTAT, 2014). The change in production is about 65% over 1991 base figures, and both gains in area (40%) and productivity (41%) have contributed to these remarkable increases in production. Over 30% of the total cowpea area is currently under improved varieties, with adopters representing an estimated 50% of the cowpea‐growing households.

## 7 FUTURE PROSPECTS AND ROADMAP FOR GRAIN LEGUMES BREEDING

The breeding efficiency and the rate of genetic gains need to be enhanced. There are opportunities to reduce time required in development of a cultivar by integrating genomics‐assisted breeding approaches and rapid generation advancement, thereby enhancing genetic gains. Recent years have witnessed extraordinary growth in development of genomics resources (structural and functional molecular markers, integrated genetic map, mapping of genes/quantitative trait loci, whole‐genome sequencing). New molecular breeding approaches like genomic selection (GS) for enhancing genetic gains for development of elite breeding lines for more complex traits could be employed. New advances in phenotyping and statistical tools need to be explored and adopted to enhance genetic gains. The data collection, management and sharing tools such as the Breeding Management System (BMS) of Integrated Breeding Platform (IBP) offer opportunity for improving efficiency of breeding programmes.

Capacity of NARS institutions should be built for modern breeding approaches so that farmers are able to more completely leverage the advances brought by these technologies. Breeding efficiency, capacity and quality of the breeding programmes will be significantly improved if breeders adopt modern breeding best practices sustainably. Legume breeders should strive towards improving targeting, speed, scale, efficiency, quality (control, precision and accuracy) according to their unique characteristics and resources.

To improve targeting, capacity of the breeding programmes should be improved to develop formal product profiles for the key varieties needed for each region, prioritize traits and rationalize resource allocation to priorities and to allocate testing to best align with target environments and markets. The breeding speed could be faster if the programmes have capacities to obtain additional generations per year, implement rapid single seed descent (SSD) workflow with appropriate recycling of elite parents in the pipeline and establish a calendar workflow by month with key activities to achieve rapid cycling around the year. Throughput could be increased with similar investment if the breeders make more crosses, handle larger populations and evaluate more plots at more sites.

Adoption of digital tools like the BMS and molecular marker systems will help to achieve greater scale. Use of modern high‐throughput phenotyping and genotyping protocols and platforms would greatly increase throughput and allow greater scale and efficiency. Designs that optimize plot size, number of locations and reps per location, increased mechanization and automation (plot threshers, seed cleaners, seed counters, bar coding, electronic data capture) and dissemination models that are rapid and that support rapid varietal replacement would together enhance efficiency. Breeders should be able to broaden genetic base by greater use of genetic diversity, either natural or artificial.

Processes that improve precision and accuracy of data and data handling such as improved electronic capture, bar coding of sample would greatly enhance data quality. Improved experimental and statistical designs and methods (e.g. statistical removal of field trends in trials) through the BMS, genotyping and analytics for quality control of parents and crosses and better trial site land management and other practices to reduce experimental error/increase heritability would all together improve product quality.

It is important for breeders to track pipeline metrics by test stage—numbers of crosses, lines established per cross, lines in initial evaluation and yield trials to track improvements made. Other data quality metrics of these trials should be tracked for trends; for example, CV% should be decreasing. It is also important for breeders to track genetic progress and genetic gains of the breeding programme. Breeding progress measures the progress achieved in improving a target trait in a breeding programme and is measured by comparing the finished lines or varieties selected from one year to next for that trait. Genetic gains is achieved when the selected plant(s) has a better combination of genes (i.e., genotype) that control the traits of interest than the unselected plant(s).

Traits that reduce drudgery through mechanized farm operations (chickpea), herbicide tolerance for chemical weed control, provision of fuel wood and fodder from bushy varieties (pigeonpea), improved and efficient postharvest handling such as simple shelling (groundnut), threshing, dehulling and dal making (chickpea/pigeonpea), early‐maturing sweet varieties for sprout‐making and green pea consumption would work together in the favour of women and youth. Many legumes are sold in the market unprocessed in the form of fresh green pods (common bean, pigeonpea, faba bean and groundnut), fresh green grains (chickpea, pigeonpea, faba bean, field pea, vegetable soybean, faba bean) and fetch better prices in these forms than dried grain. High returns to investment are expected when production is linked to new opportunities for processing and marketing (Bugusu & Marshall, 2011).
